# The Effect of Rhizoboxes on Plant Growth and Root: Shoot Biomass Partitioning

**DOI:** 10.3389/fpls.2019.01693

**Published:** 2020-01-17

**Authors:** Tereza Mašková, Adam Klimeš

**Affiliations:** ^1^ Department of Botany, Faculty of Science, Charles University in Prague, Prague, Czechia; ^2^ Department of Functional Ecology, Institute of Botany, Academy of Sciences of the Czech Republic, Třeboň, Czechia

**Keywords:** experimental container, nutrient supply, plant growth, 2D pot shape, rhizobox, root:shoot biomass partitioning

## Abstract

Various types of flat rhizoboxes aid in root visualization and tracking in experiments where the focus is upon root system growth and development. While size of the pot is known to affect experiments, nothing is known about the impact of rhizoboxes—not only their volume, but also their shape might affect root and shoot growth. Therefore, we investigated how rhizoboxes change plant biomass and root:shoot biomass partitioning. We compared biomass and root:shoot ratio of plants growing in the pots with different geometry—usual three-dimensional, cuboid plant pots and flat two-dimensional rhizoboxes about the same volume. We used two different nutritional treatments (deionized water and additional nutrients) for investigating whether the nutrient availability in the substrate changed the impact of rhizoboxes on plant growth. We used 15 species for the generalizability of our results across the phylogenetic tree. Proportional investment of plants into roots was similar in usual pots and in rhizoboxes. This pattern was stable across nutrition treatments and across species. Further, we found no differences in total biomass of plants between pot type within nutrient treatments. With added nutrients, the plants had a higher biomass and lower root:shoot ratio compared to treatments without nutrient addition. Thus, species can be safely compared when grown in the rhizoboxes; rhizoboxes did not affect root system growth comparisons among species and nutrient levels. Also, they did not affect plant growth in terms of total biomass.

## Introduction

Laboratory experiments are a routine tool in modern plant ecology for uncovering and understanding basic processes about plant behavior, especially under different environmental settings or for multilevel experiments. For study of root system behavior and plasticity, rhizoboxes are usually used because they allow continuous observation of uninterrupted root growth compared to plant cultivation by other means, e.g., pots or in the field, which only allow samples to be taken, hence disturbing or killing the individual plants under study.

Rhizoboxes can vary greatly in shape and size—from a petri dish (Gross et al., 1992) through a half-cylinder (Falik et al., 2005) to flat rectangular pots (Marschner and Römheld, 1983; Schmidt et al., 2018; Mašková and Weiser, 2019). No matter the shape, during cultivation, rhizoboxes are inclined so that the root system is forced to grow along the flat front transparent wall. This pronouncedly deforms the space that the roots can occupy. Instead of three dimensions, the root system is forced to grow in essentially two. This reduction of dimensionality allows both full tracking of root system growth and also eases analysis of the root system than is the case in three-dimensional space.

It is silently assumed that this simplification forces the roots to grow along one pot side and does not fundamentally affect the behavior of the plant root system—for example, if plants allocate more biomass into their root systems under some conditions than others, it would do so when the roots are grown either in regular pots or in the rhizoboxes. On the other hand, it is known that the size of experimental pots can affect the results of an experiment (Poorter et al., 2012). It even turns out that the shape of regular pots (i.e., the ratio of their height and diameter; McConnaughay et al., 1993), the material the pots are made of (Bunt and Kulwiec, 1970), or their color (Markham et al., 2011) can slightly affect plant growth, mainly through their effect on soil and root temperature. But it is not known how the critical change of pot shape in the rhizoboxes affects plant growth and behavior. Plants in rhizoboxes may have a large fraction of their roots growing at the pot boundary, with all kinds of secondary consequences, among others a changed root:shoot ratio (Herold and McNeil, 1979).

The main goal of this study is to show whether growth and behavior (measured as total biomass and root:shoot biomass partitioning) of plants growing in regular three-dimensional pots and flat “two-dimensional” pots of the same volume differ. Both total biomass and root:shoot biomass partitioning are closely related to soil nutrient availability (Chapin et al., 1987); therefore, we used two different levels of nutrient supply. We worked at an interspecific level to generalize our results across the phylogenetic tree.

## Materials and Methods

### Plant Cultivation

We grew plants in two differently shaped pots: *regular pots*—square “three-dimensional” pots (upper size 7 × 7 cm, bottom size 5 × 5 cm, height 8 cm, the volume 290.7 cm^3^); and *flat pots*—rhizoboxes typically used for visualization of root systems (inner dimension 19.5 × 15 × 1 cm h × w × d, the volume 292.5 cm^3^) consisting of PVC boards glued together with silicone sealant, with one of the larger sides transparent. The transparent side was covered by a non-transparent panel during plant growth.

We chose perlite (expanded amorphous volcanic glass) as a substrate for cultivation of the plants. It provides good aeration and leaches practically no nutrients so we were able to control all nutrients by watering. We used two different nutrient treatments to assess whether the nutrient availability in the substrate changes the effect of the pot shape. We used universal fertilizer solution (Wuxal Super; manufactured by AGLUKON Specialdünger GmbH & Co.KG, Düsseldorf; N:P:K = 8:8:6; **Supplementary Table 1**) diluted in water to a 0.1% volumetric concentration, and pure deionized water. Half of the individuals in each type of pot were subjected to each nutrient treatment.

We used 15 common central European herbaceous species spread over the phylogenetic tree (**Supplementary Table 2**). All seeds were acquired from a commercial supplier (Planta Naturalis, www.plantanaturalis.com).

All seeds were germinated individually in Petri dishes on filter paper moistened with 3 ml of the respective fertilizer treatment solution. Pure deionized water was added as needed so that the paper remained moist throughout. The Petri dishes were kept in a growth chamber (Adaptis A 1000 with TC kit, Conviron, Canada; light intensity 225 μmol/cm^2^/s at a distance of 12.5 cm from the light source) under the following diurnal temperature regime: 20°C for 12 h during the day and 10°C for 12 h during the night. On the day the radicle emerged through the seed testa, the seed was transferred into its own individual pot. Cultivation of the plants in pots took place in the same growth chamber used for germination and under the same temperature and light settings. The relative air humidity was set to 50% during the day and 70% during the night. The pots were evenly spaced to minimize the effect of aboveground competition (plant density were 220 and 240 individuals per m^2^ for the *regular pots* and the *flat pots*, respectively).

Initially, we aimed to have 6 replicates per species per nutrient treatment in the *regular pots* and 10 replicates per species per nutrient treatment in the *flat pots*. This would have led to 32 pots per species and 480 pots total. Due to technical reasons (not all plants survived transplantation), the actual number of pots per species per nutrient treatment ranged from 5 to 6 for *regular pots* and 5 to 13 for *flat pots*.

We harvested plants after 4 weeks of growth (at this point, plants had true leaves or lateral roots, root system were in case of rhizoboxes touching the transparent side-wall) and separated their root and shoot biomass at the boundary between epicotyl and hypocotyl. We extracted roots carefully from the perlite without using water; if necessary, we crushed the perlite piece softly so that the roots remain intact. We dried root and shoot parts at 65°C for 2 days and weighed them (scale accuracy: 0.1 mg). We calculated total biomass (as sum of root and shoot biomass) and root:shoot ratio. We used total biomass and root:shoot ratio as an approximation of plant growth dynamics because it is known that other proxies of growth (e.g., SLA) are not correlated with pot size (Poorter et al., 2012). In cases where root biomass was below the detection limit, we used half of the limit (0.05 mg) as root biomass in calculation of the root:shoot ratio. We have also repeated the analysis with observations below the detection limit excluded.

### Data Analysis

To assess the effect of pot type on total biomass and root:shoot ratio, we ran phylogenetic hierarchical linear models in a Bayesian framework. Response [natural logarithm of total biomass (g) and of root:shoot ratio] was modelled for each species as:

$$Resp\;\sim \;Normal\left(\alpha +\beta_{1}*P+\beta_{2}*N+\beta_{3}*P*N,\;\sigma \right)$$

where *P* is the type of pot, *N* is the nutrient level, *σ* is a standard deviation parameter, and *α*, *β* are species-specific parameters. We modelled *α*, *β* as:

$$par\;\sim \;MultivariateNormal\left(\mu_{par},phy*\lambda_{par}*\gamma_{par}\right)$$

where *par* is the parameter *α, β_1_, β_2_*, or *β_3_. μ_par_* is a mean parameter (specific for each *α* or *β*) and *phy* is a phylogenetic variance–covariance matrix, *λ* is a parameter that multiplies only off-diagonal elements of the variance–covariance matrix [thus Pagel's *λ* (1999)], and *γ* is a parameter (multiplies all elements of the matrix) specific for each *α* and *β*. The parameter *λ* was constrained to interval [0,1] and *γ* was constrained to be larger than 0.

For parameters we used uninformative Cauchy-distributed (with mean 0 and scale 5) priors, apart from parameters *α* and *λ* where we used uniform priors (on R for *α* and on interval [0,1] for *λ*). Models were evaluated using Hamiltonian Monte Carlo with a No-U-Turn sampler (Hoffman and Gelman, 2014) with 4 chains, 4,000 iterations each, 2,000 of them as a warm-up phase. Convergence was checked using Ȓ statistics (Ȓ was for all parameters within interval [0.99,1.01]; Gelman and Rubin, 1992). Analyses were done in R (R Core Team, 2016) using package *rstan* (version 2.17.3; Carpenter et al., 2017; Stan Development Team, 2018). Phylogenetic information was taken from Durka and Michalski (2012).

## Results

**Figure 1:**
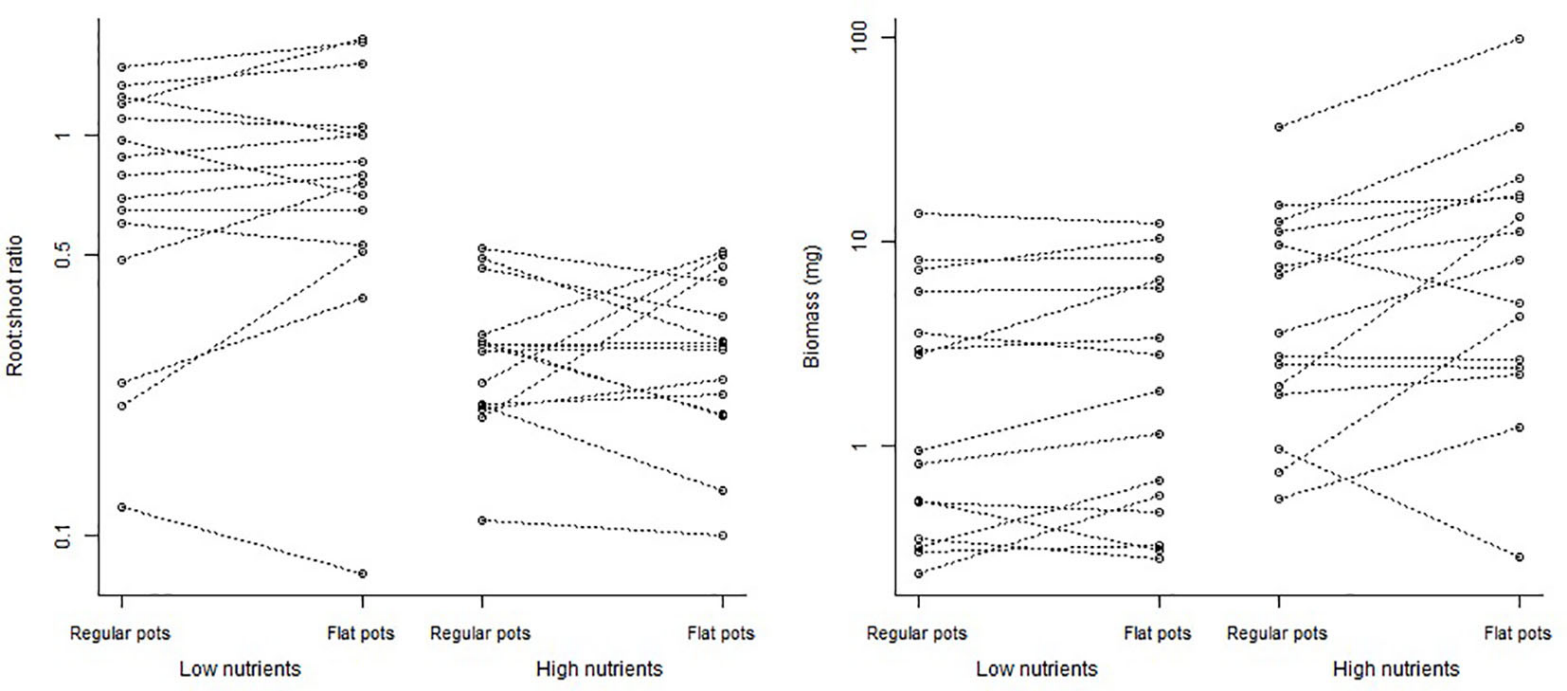
Root:shoot ratio and total biomass of plants growing in the *flat pots* and *regular pots.* Points indicate mean values for different species per nutrient supply; lines connect individual species. Root:shoot ratios and total biomass were logarithmically transformed. For species specific information, see **Supplementary Figure 1**.

Total biomass of plants ranged from 0.1 to 130 mg and from 0.1 to 59 mg in the *regular pots* and *flat pots,* respectively. Plants under high nutrient treatment had 2.8 times more aboveground biomass [95% credible interval (1.6820, 4.6450); **Figure 1**]. The total plant biomass was not affected by type of pots [plants in flat pots had 1.2 times more biomass; 95% credible interval (0.8338, 1.8340)] in both nutrition treatments [1.4 posterior mean of the interaction parameter; 95% credible interval (0.6703, 2.8205); **Table 1**].

**Table 1:** Effect of pot type and nutrient treatment on logarithm of total biomass of plants and root:shoot ratio of plants. Values in brackets are on the (natural) logarithmic scale.

Measured variable	Effect	Parameter	Posterior mean	95% credible interval
Total biomass of plants	Type of pots	μ_β1_	1.2310 (0.2078)	[0.8338, 1.8340] (-0.1818, 0.6065)
	**Nutrients**	**μ_β2_**	**2.8075 (1.0323)**	**[1.6820, 4.6450] (0.5200, 1.5358)**
	Type of pots*nutrients	μ_β3_	1.3793 (0.3216)	[0.6703, 2.8205] (-0.4000, 1.0369)
Root:shoot biomass of plants	Type of pots	μ_β1_	1.1198 (0.1131)	[0.8847, 1.4173] (-0.1225, 0.3488)
	**Nutrients**	**μ_β2_**	**0.4581 (-0.7806)**	**[0., 0.7750] (-1.3025, -0.2549)**
	Type of pots*nutrients	μ_β3_	0.9013 (-0.1039)	[0.6157, 1.3528] (-0.4849, 0.3021)

Effects that are probable to impact growth or root:shoot ratio are highlighted in bold.

The root:shoot ratio of plants ranged from 0.005 to 3 and from 0.05 to 4 in the *regular pots* and the *flat pots,* respectively. As expected, we found a lower root:shoot ratio in the higher nutrition treatment [0.46 times; 95% credible interval (0.2718, 0.7750); **Figure 1**]. Further, we found no effect of type of pot on the root:shoot biomass partitioning of plants. Our results suggested a slight tendency to invest more into the below-ground biomass in the *flat pots* [a 1.12 times larger root:shoot ratio than in the regular pots; 95% credible interval (0.8847, 1.4173); **Figure 1**]. This pattern did not differ between nutrition treatments [0.9 posterior mean of the interaction parameter; 95% credible interval (0.6157, 1.3528); **Table 1**] and exclusion of plants with root biomass below the determination limit did not influence the results either.

## Discussion

Plant growth in rhizoboxes was comparable to the growth in regular pots. This study showed that the constraints of growth of seedling root system in the rhizoboxes compared to the growth in regular pots are probably not stressful for plants and do not change their growth dynamics. However, plants growing in the *flat pots* partitioned their biomass slightly differently than the plants growing in the *regular pots* of the same volume. Although this effect was small, we think caution is necessary when interpreting the results of experiments with rhizoboxes. This effect disappeared after exclusion of the smallest plant (with root biomass below scale accuracy). Some species responded more than others with no obvious driver of this difference (for details, see **Supplementary Figure 1**). In the *flat pots*, plants tend to invest higher proportion of biomass into their root system than plants in the regular pots, probably because they have to cope with obstacles in the form of pot side. Although the volume is the same for both type of pots, in the flat pots, substrate particles are distributed further away from the rooting point. Therefore, they are accessible only with more root growth and hence there are less substrate-bond resources available to the roots of the same total length, although the exact figures may vary according to the root intake capacity and mobility of the resources in the substrate matrix. Moreover, the pot surface between pot types differs substantially. Thus, the effects of the pot boundary (Herold and McNeil, 1979; Ou, 2014) or root self-inhibition (Falik et al., 2005) could have a higher importance in the *flat pots* than in the *regular pots. S*imilar mechanisms play a role in terms of the effect of substrate texture (Semchenko et al., 2007).

Similarly, we found almost no differences in total biomass of plants between the pot types. It seems that changing pot shape into rhizoboxes does not mean a substantial difficulty for development of a root system capable of utilizing available nutrients. This supports the finding of Poorter et al. (2012), who showed that it was not pot size *per se* but rather plant mass per unit rooting volume that is relevant for plant growth. Nevertheless, it was shown that pot size affects plant growth (Keever et al., 1986; Poorter et al., 2012) and also could change the result of an experiment (Arp, 1991); we therefore recommend carefully considering the pot volume as well for experiments with rhizoboxes.

The type of pot affected total plant biomass and root:shoot biomass partitioning much less than the nutrient supply. It has been shown many times before that the nutrient supply is an important driver of plant growth and root:shoot biomass allocation in pots (Gedroc et al., 1996) and also in the field (Delgado et al., 2011), and our results confirmed the same effect of nutrient availability on plant growth and development during their growth in rhizoboxes. In favor of the experiments based on rhizoboxes, the differences between usual pots and rhizoboxes were not amplified with an increased plant size induced by the fertilization. This suggests that the effects of rhizoboxes are not substantially allometric; hence, the effects might be relatively easy to predict.

## Conclusion and Perspectives

Rhizoboxes provide a great opportunity to study root system behavior and dynamics. The idea of visualization of root system architecture is not new (Marschner and Römer, 1983; Youssef and Chino, 1987; Fitter et al., 1988) but processing the data from rhizoboxes by using combination of hand measurements, computers, and scanners or cameras of that time was very time consuming, expensive, and inefficient. Since the beginning of this century, increasing computational power and decreasing cost of computers led to development of appropriate software and increase in number of studies, which were growing plants in rhizoboxes. This trend can be expected to continue because many processes hidden underground are still unknown. Moreover, many recent studies deal with how to construct rhizoboxes more cheaply and efficiently (Hylander, 2002; Schmidt et al., 2018).

Rhizoboxes are very useful tool for understanding the processes usually hidden below ground. But by their very nature, their use always means pronounced modification of natural conditions, at least a huge pot surface increase which adds to the importance of the pot boundary effect. Obviously, this enormous constraint of growth space does not allow us to observe directly what is going on below ground in absolute terms and dimensions. It is silently assumed, and our results basically confirm this, that comparisons of root system growth among species under various conditions could be done in rhizoboxes as well as in regular pots. However, comparisons within a species, or comparison of pot-based and rhizobox-based results, should be done with caution.

## Data Availability Statement

This data can be found here: https://datadryad.org/stash/dataset/doi:10.5061/dryad.67mg4q8, https://datadryad.org/resource/doi:10.5061/dryad.gv33qc2.

## Author Contributions

TM designed and performed the experiment, AK analyzed the data, TM and AK interpreted results, and TM wrote the text with contributions of AK. Both authors approved the final version of the manuscript.

## Funding

This work was supported by the Czech Science Foundation No. 19-0630S.

## Conflict of Interest

The authors declare that the research was conducted in the absence of any commercial or financial relationships that could be construed as a potential conflict of interest.
