# Supplementary material for: The Effect of Rhizoboxes on Plant Growth and Root: Shoot Biomass Partitioning
Source: Front Plant Sci. 2020 Jan 17;10:1693. doi: 10.3389/fpls.2019.01693 (PMC6978282; doi:10.3389/fpls.2019.01693)

Supplementary material:

Supplementary Table 1: Nutrient content of the fertilizer (Wuxal Super; AGLUKON Specialdünger GmbH & Co.KG, Düsseldorf) in the concentrate supplied by the manufacturer. It was diluted further to reach the highest administered concentration in the experiment (0.1% v/v). N:P:K is 8:8:6.

| % w/w |  |  | g/l |
| --- | --- | --- | --- |
| 1.935 | NH_2_ | amide nitrogen | 24 |
| 3.71 | NH_3_ | ammoniac | 46 |
| 2.258 | NO_3-_ | nitrate | 28 |
| 8 | P_2_O_5_ | phosphate | 99.2 |
| 6 | K_2_O | potassium | 74.4 |
| 0.887 | S | sulphur | 11 |
| 0.01 | B | boron | 0.124 |
| 0.004 | Cu | copper | 0.049 |
| 0.02 | Fe | iron | 0.248 |
| 0.012 | Mn | manganese | 0.148 |
| 0.004 | Zn | zinc | 0.049 |
| 0.001 | Mo | molybdenum | 0.012 |

Supplementary Table 2: Plant species used and number of individuals per type of pot and nutrition treatment in the experiment.

| Species | *Flat pots* | | | *Regular pots* | |
| --- | --- | --- | --- | --- | --- |
|  | deionized water | nutrient supply | deionized water | | nutrient supply |
| *Agrimonia eupatoria* | 12 | 13 | 6 | | 6 |
| *Anchusa officinalis* | 11 | 10 | 6 | | 6 |
| *Campanula glomerata* | 11 | 8 | 6 | | 5 |
| *Campanula trachelium* | 11 | 5 | 6 | | 6 |
| *Centaurea cyanus* | 10 | 10 | 6 | | 6 |
| *Dianthus deltoides* | 10 | 10 | 6 | | 6 |
| *Filipendula vulgaris* | 9 | 10 | 6 | | 6 |
| *Inula britannica* | 10 | 7 | 6 | | 6 |
| *Lathyrus vernus* | 11 | 9 | 6 | | 6 |
| *Lepidium campestre* | 11 | 11 | 6 | | 6 |
| *Lithospermum arvense* | 10 | 10 | 6 | | 6 |
| *Lotus corniculatus* | 12 | 11 | 6 | | 5 |
| *Lychnis viscaria* | 10 | 9 | 6 | | 6 |
| *Nigella arvensis* | 10 | 8 | 6 | | 6 |
| *Sisymbrium officinale* | 10 | 10 | 6 | | 6 |

Supplementary Fig. 1: Root:shoot ratio of 15 species in *flat pots* (2D) and *regular pots* (3D) and two levels of nutrients. Geometric mean values for each species and treatment are visualized by bars. Error bars denote standard errors. Species are sorted by mean proportional strength of their response to *flat pots* (in comparison with the regular ones) – species with highest increase of root:shoot ratio are on the left.


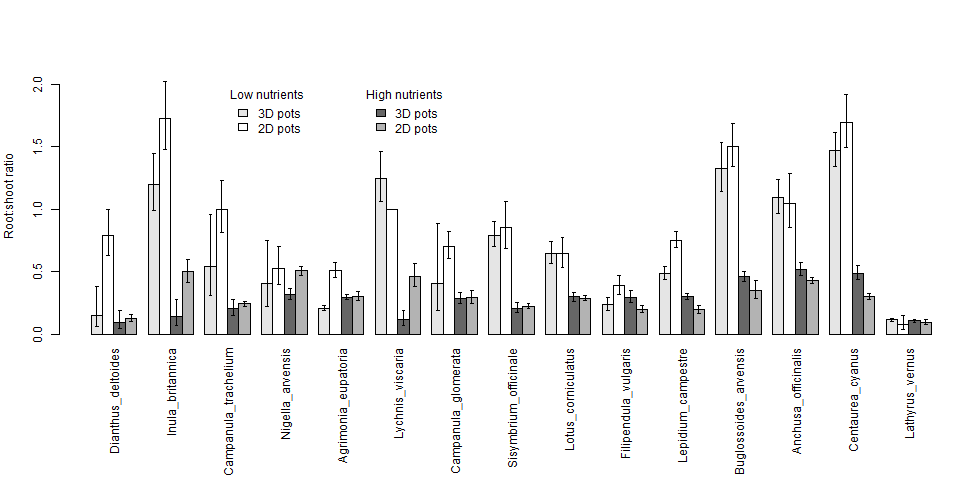

Supplement: Supplementary file 1 [file Table_1.docx]
